# Capsular synovial metaplasia mimicking silicone leak of a breast prosthesis: a case report

**DOI:** 10.1186/1752-1947-2-277

**Published:** 2008-08-15

**Authors:** Sarah Krishnanandan, Ali Abbassian, Anup K Sharma, Giles Cunnick

**Affiliations:** 1 SpR in Accidents and Emergency, Kingston Hospital, Kingston, Surrey, KT2 7QB, UK; 2 SpR in Trauma and Orthopaedics, Mayday University Hospital, Croydon, CR7 7YE, UK; 3 Department of Breast Surgery, St. George's Hospital, Tooting, London, SW17, UK; 4 Wycombe General Hospital, Queen Alexandra Road, High Wycombe, Bucks, HP11 2TT, UK

## Abstract

**Introduction:**

Synovial metaplasia around a prosthesis and in particular around silicone breast implants has been noted by various investigators, but has unknown clinical significance. We report on a patient where a large amount of synovial fluid mimicked rupture of an implant. We believe this to be an unusual clinical presentation of this phenomenon. Review of the English language literature failed to identify a comparable case.

**Case presentation:**

A 25-year-old woman had undergone bilateral breast augmentation for cosmetic reasons. One implant was subsequently subjected to two attempts at expansion to correct asymmetry. The patient was later found to have a large quantity of viscous fluid around the port of that same prosthesis. Histological assessment of the implant had consequently confirmed capsular synovial metaplasia. This had initially caused the suspicion of a silicone 'bleed' from the implant and had resulted in an unnecessary explantation.

**Conclusion:**

Capsular synovial metaplasia should be ruled out before the removal of breast implants where a leak is suspected. Manipulation and expansion of an implant may be risk factors for the development of synovial metaplasia.

## Introduction

Synovial metaplasia around prostheses is regarded as a transitional phenomenon [1]. We describe the case of a patient in whom, at the time of removal of a prosthetic port, the quantity of viscous fluid produced as a result of metaplasia caused us to suspect that a silicone bleed had occurred. This resulted in what later appeared to have been the unnecessary explantation of her implant.

## Case presentation

A fit and healthy 25-year-old Caucasian woman presented with asymmetry and gross bilateral tubular deformity of the breasts. Subsequently, a bilateral breast reconstruction with 350 cc Becker™ (Mentor, UK) implants was performed. At the time of surgery, 200 ml of saline was used to inflate both implants. The left implant was further inflated 1 and 5 months later using 80 ml of normal saline on both occasions, to optimize symmetry.

**Figure 1:**
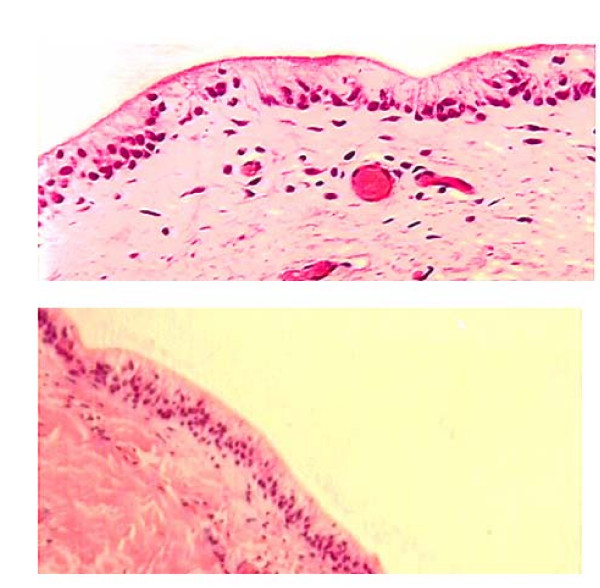
**Capsular synovial metaplasia.** Sections from the cavity show the surface of the capsule lined by fibrohistiocytic cells. The nuclei of these cells are basally oriented and are polarized perpendicular to the cavity surface. The interface between the capsule and implant space is smooth. The overall features are indicative of synovial metaplasia.

One year later, both ports were removed as a day case procedure. At that time, the right port was removed without complication. However, the left port was surrounded by a viscous fluid simulating implant rupture. A silicone gel bleed was suspected and another operation was planned where the left prosthesis was replaced with another permanent implant. The fluid and a sample of the periprosthetic capsule were sent for histological review. Histological examination revealed that the fluid was synovial fluid. The sample of capsule was found to be fibroadipose tissue, composed of fibrin-organizing histiocytes, lymphocytes and multinucleate giant cells. The capsule had undergone synovial metaplasia, which explained the presence of synovial fluid around the left implant. A typical histological appearance of synovial metaplasia is shown in Figure 1.

## Discussion

Synovial metaplasia was first described by Brody and White [2] following their studies on implanted silicone joints in chickens. Another study of 85 patients with breast prostheses showed that the incidence of synovial metaplasia was 40% and that this condition was not as rare as had been previously suspected [1].

A number of theories have been suggested to explain this phenomenon. One study suggested that the tissue reaction was a response to implants with a textured surface rather than a smooth surface [3]. In contrast, Ko *et al*. [1] suggested that the occurrence of synovial metaplasia did not correlate with the implant type. Instead they postulated that implant age may be a significant factor. The incidence had been shown to decrease with the age of the implant, suggesting that it may be a transitional finding in capsular maturation. This is in contrast to one report of synovial metaplasia that had presented with breast firmness and pain 26 years following implantation [4].

Another hypothesis suggests that mechanical stress may influence the development of synovial metaplasia. In one study [5], the bone-cement interface of loose hip prostheses, which is under considerable mechanical stress, was shown to undergo synovial metaplasia. Mechanical interference has also been associated with its development in the skin [6]. Mechanical stress may influence the development of synovial metaplasia in breast implants because of repeated surgery, expansion of the implants, the pendulous movement of the breasts or with chest wall muscle activity. Synovial metaplasia secretes lubricating factors and this may be beneficial for the reduction of capsular contracture. In one report, synovial metaplasia occurred after multiple manipulations and tissue expansions [7]. The investigators believed that this played an important role in the development of the metaplasia. The mechanical interference theory may explain the findings in our patient. The left prosthesis was expanded on two occasions, whereas the right prosthesis was not expanded at all. Synovial fluid was only macroscopically evident on the left side.

## Conclusion

Silicone breast implants that are suspected of a leak should be assessed by histological examination of the fluid to rule out synovial metaplasia. This is particularly important if the implant has been subjected to expansions or manipulations. The clinical significance of synovial metaplasia is uncertain, however, increased awareness of this phenomenon by surgeons may reduce the unnecessary explantation of perfectly intact prostheses. If a leak is found to be due to synovial metaplasia, a period of observation and delay in explantation is advised as this may well be a transitional phenomenon.

## Consent

Consent could not be obtained as the patient was untraceable. However, we believe the article contains a worthwhile clinical lesson which could not be made as effectively in any other way. The risk of identification of the patient is minimized by measures designed to prevent the identity of the patient being revealed either to others or to the patient's relatives. We expect the patient and their next of kin would not object to the publication of this case.

## Competing interests

The authors declare that they have no competing interests.

## Authors' contributions

SK and AA were involved in the literature search, writing up of the case and preparing the revision. AS and GC managed the clinical care of the patient as well as assisting in writing the manuscript.
